# Assessing Complex Emergency Management with Clinical Case-Vignettes: A Validation Study

**DOI:** 10.1371/journal.pone.0138663

**Published:** 2015-09-18

**Authors:** Anne Rousseau, Patrick Rozenberg, Philippe Ravaud

**Affiliations:** 1 Department of Obstetrics and Gynecology, Poissy-Saint Germain Hospital, Poissy, France; 2 INSERM U1153 Research Unit, Paris Descartes-Sorbonne Paris Cité University, Paris, France; 3 Research unit EA 7285, Versailles-St Quentin University, Saint Quentin en Yvelines, France; 4 Assistance Publique-Hôpitaux de Paris, Centre d’Epidémiologie Clinique, Hôpital Hôtel-Dieu, Paris, France; University of Pittsburgh, UNITED STATES

## Abstract

**Objective:**

To evaluate whether responses to dynamic case-vignettes accurately reflect actual practices in complex emergency situations. We hypothesized that when obstetricians were faced with vignette of emergency situation identical to one they previously managed, they would report the management strategy they actually used. On the other hand, there is no reason to suppose that their response to a vignette based on a source case managed by another obstetrician would be the same as the actual management.

**Methods:**

A multicenter vignette-based study was used in 7 French maternity units. We chose the example of severe postpartum hemorrhage (PPH) to study the use of case-vignettes for assessing the management of complex situations. We developed dynamic case-vignettes describing incidents of PPH in several steps, using documentation in patient files. Vignettes described the postpartum course and included multiple-choice questions detailing proposed clinical care. Each participating obstetrician was asked to evaluate 4 case-vignettes: 2 directly derived from cases they previously managed and 2 derived from other obstetricians’ cases. We compared the final treatment decision in vignette responses to those documented in the source-case by the overall agreement and the Kappa coefficient, both for the cases the obstetricians previously managed and the cases of others.

**Results:**

Thirty obstetricians participated. Overall agreement between final treatment decisions in case-vignettes and documented care for cases obstetricians previously managed was 82% (Kappa coefficient: 0.75, 95% CI [0.62–0.88]). Overall agreement between final treatment decisions in case-vignettes and documented care in vignettes derived from other obstetricians’ cases was only 48% (Kappa coefficient: 0.30, 95% CI [0.12–0.48]). Final agreement with documented care was significantly better for cases based on their own previous cases than for others (p<0.001).

**Conclusions:**

Dynamic case-vignettes accurately reflect actual practices in complex emergency situations. Therefore, they can be used to assess the quality of management in these situations.

## Introduction

The first step in improving clinical practices is assessing their quality, preferably by a simple, feasible, and accurate method. Several methods have been used: standardized patients (trained actors), high fidelity simulation, chart abstraction, clinical audits, and clinical case vignettes. Clinical vignettes are short, clear texts that describe realistic clinical situations so that physicians can assess identical scenarios. Most often, vignettes have been used to survey practices, or to assess opinions or preferences [1–4]. Vignettes are intended to assess both physicians’ knowledge and their actual practices [5,6]. Peabody et al [7,8] have concluded that vignettes are a valid tool for measuring the quality of clinical practice, compared with standardized (actor) patients or chart abstraction. They measure quality of care better than chart abstraction does. Vignettes are easy to use and more cost-effective than standardized patients, high fidelity simulation, or even clinical audits [7–10]. Moreover, it is easier to control case-mix variation in vignettes than in data sets. They have thus been widely used across countries, health care systems, and specialties [11–13].

Case-vignettes have been used and validated to analyze practices such as screening, diagnosis, care, assessment of prognosis, and ethical decision making [3,5,6,14–17]. They have not, however, been validated to assess the complex management strategies often observed in emergency situations. Dynamic multistage vignettes can be constructed to assess clinical practices in such situations. The objective of our study was to determine whether such vignettes accurately reflect what physicians do in real complex emergency situations.

We focused on the situation of severe postpartum hemorrhage (PPH) because it is a common, complex emergency situation that occurs in about 1 to 2% of deliveries in developed countries [18,19].

## Materials and Methods

This multicenter cross-sectional study took place in October to November 2012. Our purpose was to determine whether responses to dynamic vignettes reflect actual practices in managing complex PPH. We hypothesized that when obstetricians were faced with a vignette of a situation of severe PPH identical to one they had previously managed, they would report the management strategy they had actually used. Conversely, we hypothesized that in cases not identical to those they had handled, they would not reproduce a strategy identical to that of the other obstetrician who did manage it. The study was intentionally conducted independently of quality of practices because our aim was simply to verify that obstetricians use the same practices in actual situations as they report in vignettes.

### Vignette Construction

Vignettes were developed by abstracting information from patient files. We retrospectively selected patient files from 7 maternity units in the Paris area—6 public university hospitals, and 1 public non-university hospital. We identified the names and number of senior obstetricians in each unit and then reviewed each hospital’s birth register to allow us to select the first 2 cases (with complete information) of severe PPH managed by each senior obstetrician, from January 2010 through December 2011. PPH was defined as severe if women received one or more of the following treatments: transfusion, intrauterine tamponade, pelvic vessel ligation, compressive suture, hysterectomy, arterial embolization, or transfer to an intensive care unit.[20] In all, we reviewed 90 medical files; 22 were excluded because of missing data, and 2 others because the obstetrician no longer worked in the unit. Accordingly, we developed 66 vignettes from the 66 complete files for 33 obstetricians.

**Fig 1:**
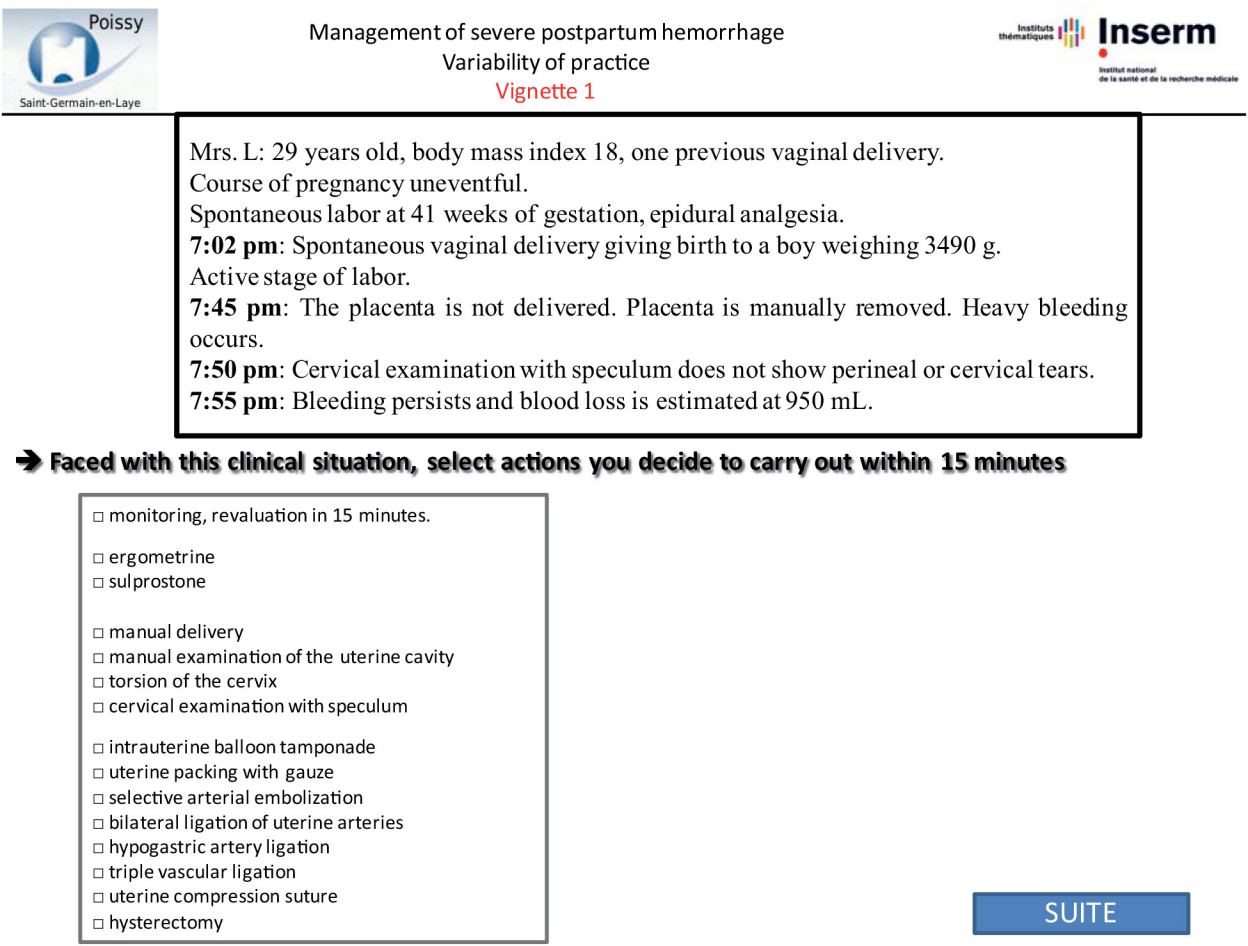
Step 1 of sample vignette. This figure corresponds to screenshot of the website: first step of vignette with closed-ended question.

**Fig 2:**
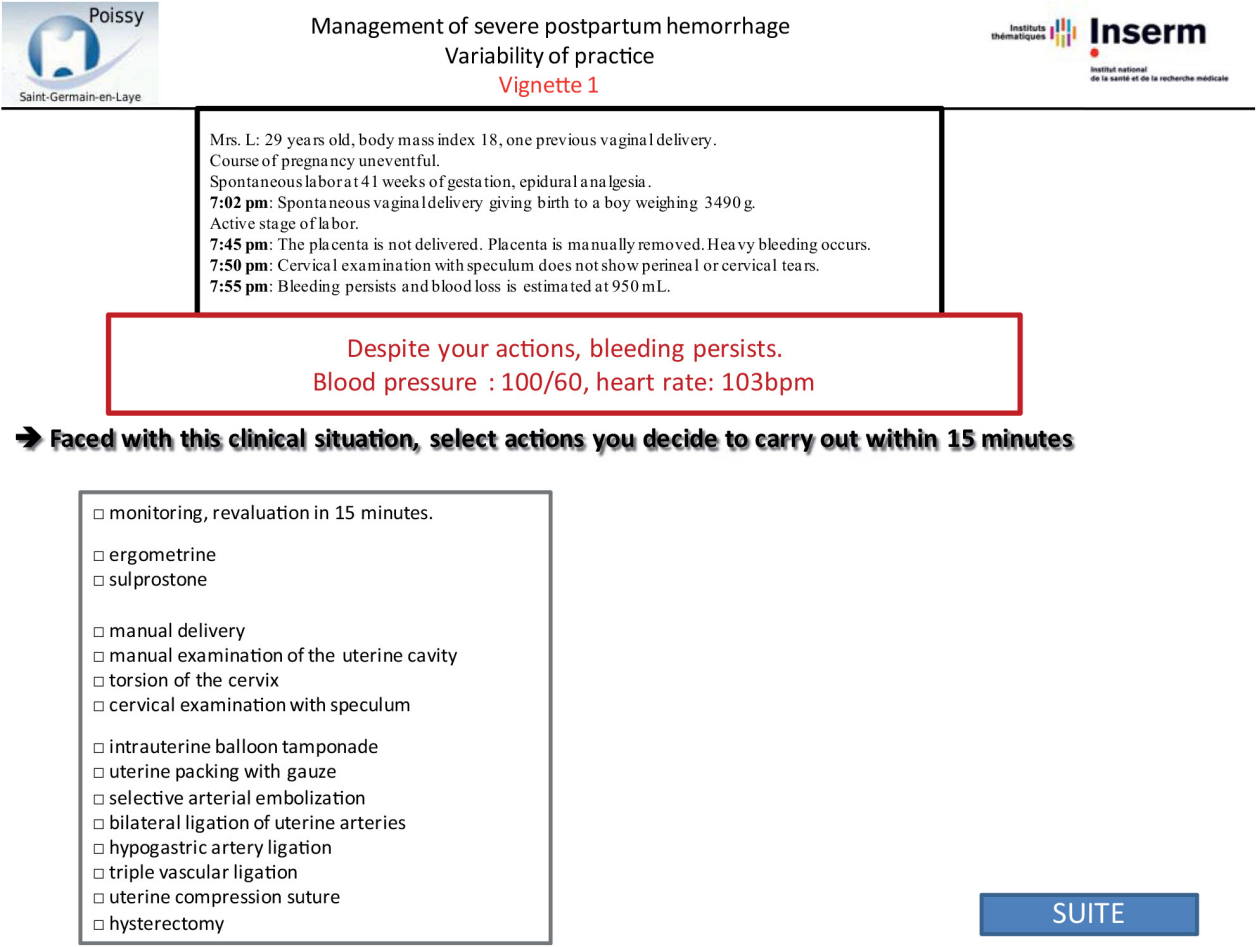
Step 2 of sample vignette. This figure corresponds to screenshot of the website: second step of the same vignette with the same closed-ended question.

**Fig 3:**
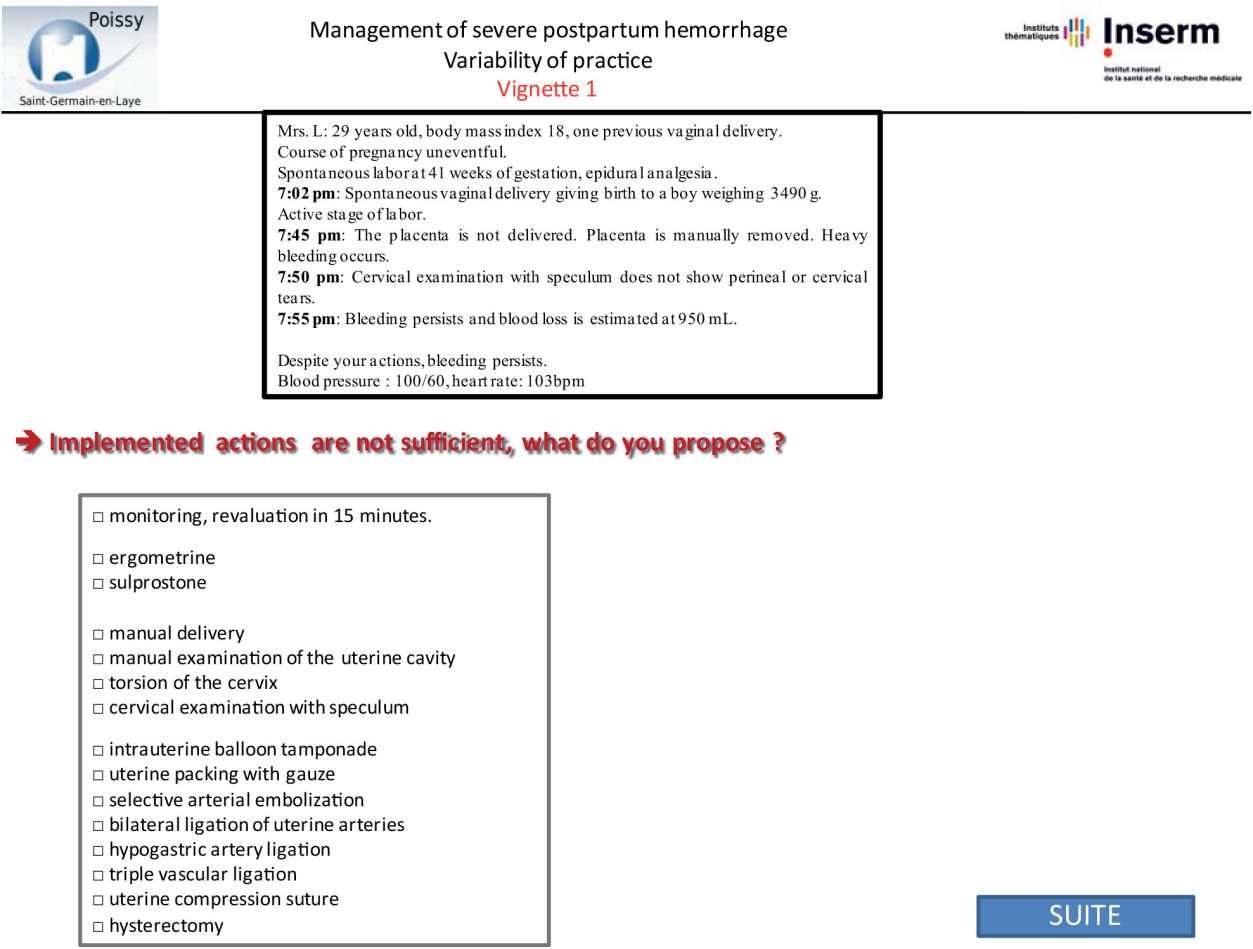
Step 3 of sample vignette. This figure corresponds to screenshot of the website: third and last step of the same vignette with the same closed-ended question.

Vignettes were developed by abstracting the following data from patient files: patient medical history and information about the pregnancy, labor, delivery, and PPH. All data that might identify the specific situation were changed. We designed the vignettes to include several steps re-creating the course of the PPH, for its management often requires several successive steps to control bleeding. For each step of the vignette, we described a stage of the postpartum course, including bleeding, maternal condition, and response to proposed treatment (Figs 1–3). We used the same standardized format for all vignettes. Each clinical vignette was pretested by an obstetrician and a midwife to check clarity and brevity.

### Survey Administration

The 33 senior obstetricians blinded to the hypothesis were invited by email to participate in the survey at a specially constructed website. The email explained that this was a pilot study assessing variability in PPH management, and a link to the questionnaire was provided at the end of the message. By following the link to the questionnaire and completing it, they provided informed consent. Obstetricians who did not complete the survey received two gentle email reminders 2 weeks apart [21].

Each questionnaire included 4 clinical vignettes: 2 were based on their own previous cases and 2 on other obstetricians’ cases randomly assigned from the remaining 64 cases. The obstetricians were not told that any of these cases were based on real cases, let alone their own. Instead, at the end of the questionnaire, we asked the following 3 questions to evaluate memory bias: Do you think this clinical vignette is derived from a real case? Please select two among these 4 case-vignettes that correspond most closely to situations you have previously managed. Do you think you have changed your PPH management during the last 2 years? The order of presentation of the 4 case vignettes was randomized. Obstetricians were asked how they would manage each step of the case vignettes. We used the same closed-ended questions with the following set of items in a multiple-choice format for answers for each step [22]: monitoring, manual delivery of the placenta, manual examination of the uterine cavity, cervical examination with speculum, torsion of the cervix, use of ergometrine or sulprostone, intrauterine tamponade, selective arterial embolization, bilateral ligation of uterine arteries, hypogastric artery ligation, triple vascular ligation, uterine compression sutures, and hysterectomy (Figs 1–3). After responding to each step, obstetricians could not return to the previous step to change their answer.

### Main Outcomes: Agreement between Vignette Response and Documented Care

Agreement was assessed according to 2 methods. The first method, which we called final agreement, allowed us to determine if the result at the end of the vignette response was the same as at the end of the documented care. The second method, which we called sequential agreement, allowed us to explore the successive steps of management between the vignette response and the documented care, even in cases where the final action was the same.

#### Final agreement

**Fig 4:**
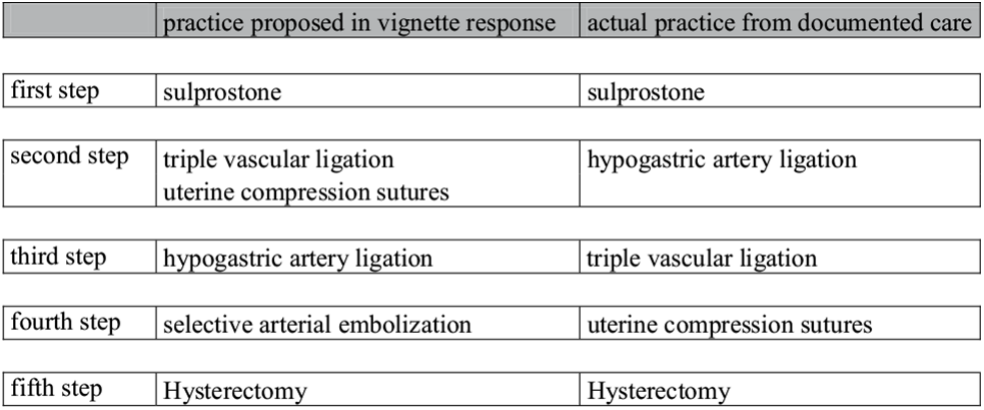
Example of final agreement and sequential agreement. In this example final agreement was high because the final treatment proposed at the last step of the vignette response was identical to the final treatment administered at the end of the vignette source situation in the documentation: hysterectomy. However sequential agreement was low because the management sequence was dissimilar between that proposed in the vignette response and that in the vignette source situation from the documentation: selective arterial embolization was added as a potential vignette response.

Final agreement was defined as agreement between the final treatment proposed at the last step of the vignette response and final treatment administered at the end of the actual situation, based on the case documentation. We evaluated final agreement, both for the cases the obstetricians had previously managed and those managed by other obstetricians (Fig 4).

The 5 potential choices for final treatment were use of sulprostone, intrauterine tamponade, selective arterial embolization, hysterectomy, and other surgical treatment.

#### Sequential agreement

Sequential agreement was defined as agreement between the management sequence proposed in the vignette responses and the management sequence of the source situation, as documented in the initial records, taking into account the different decisions made and their order throughout the sequence. Sequential agreement was determined by expert consensus: 2 specialists assessed sequential agreement for each situation as low or high. It was low if the initial or second step differed, even if the final step was the same. When the two assessors disagreed, a third obstetrician was consulted. The specialists determined consensus for every situation. They did not know which situations corresponded to cases previously managed by the obstetrician and those managed by other obstetricians. We compared sequential agreement between the cases the obstetricians had previously managed and the cases managed by other obstetricians (Fig 4).

### Ethics Statement

Our institutional review board (Comité de Protection des Personnes Ile de France Paris- XI) approved this study on September 13, 2012, as number 12066.

We obtained the Head of Department consent to consult patient files. Patient file information was anonymized and de-identified prior to analysis. All data that might identify the specific situation were changed.

Participants were all senior obstetricians who completed a questionnaire about how they would respond to 4 clinical vignettes; they were invited to participate by email. By clicking on the survey link and completing the questionnaire, they provided informed consent to participate. Participants were informed about the purpose of the study after completion of the study by email.

### Statistical Analysis

Data are available in S1 Table.

Qualitative variables were described with frequencies and percentages. Agreement between the final treatment decision in vignette responses and the documented care was assessed with the Kappa coefficient. Based on the standards outlined by Landis and Koch [23], a Kappa coefficient <0 was considered to be poor agreement, 0–0.20 slight, 0.21–0.40 fair, 0.41–0.60 moderate, 0.61–0.80 substantial, and 0.81–1.0 almost perfect agreement. A Chi-square test was used to compare the proportion of overall final agreement in cases the obstetricians had and not previously managed, and the proportion of low sequential agreement in each of these groups.

Tests were two-tailed and *P* <0.05 was considered statistically significant. R software version 2.14 (http://www.R-project.org, the R Foundation for Statistical Computing, Vienna, Austria) was used for the statistical analysis.

## Results

**Fig 5:**
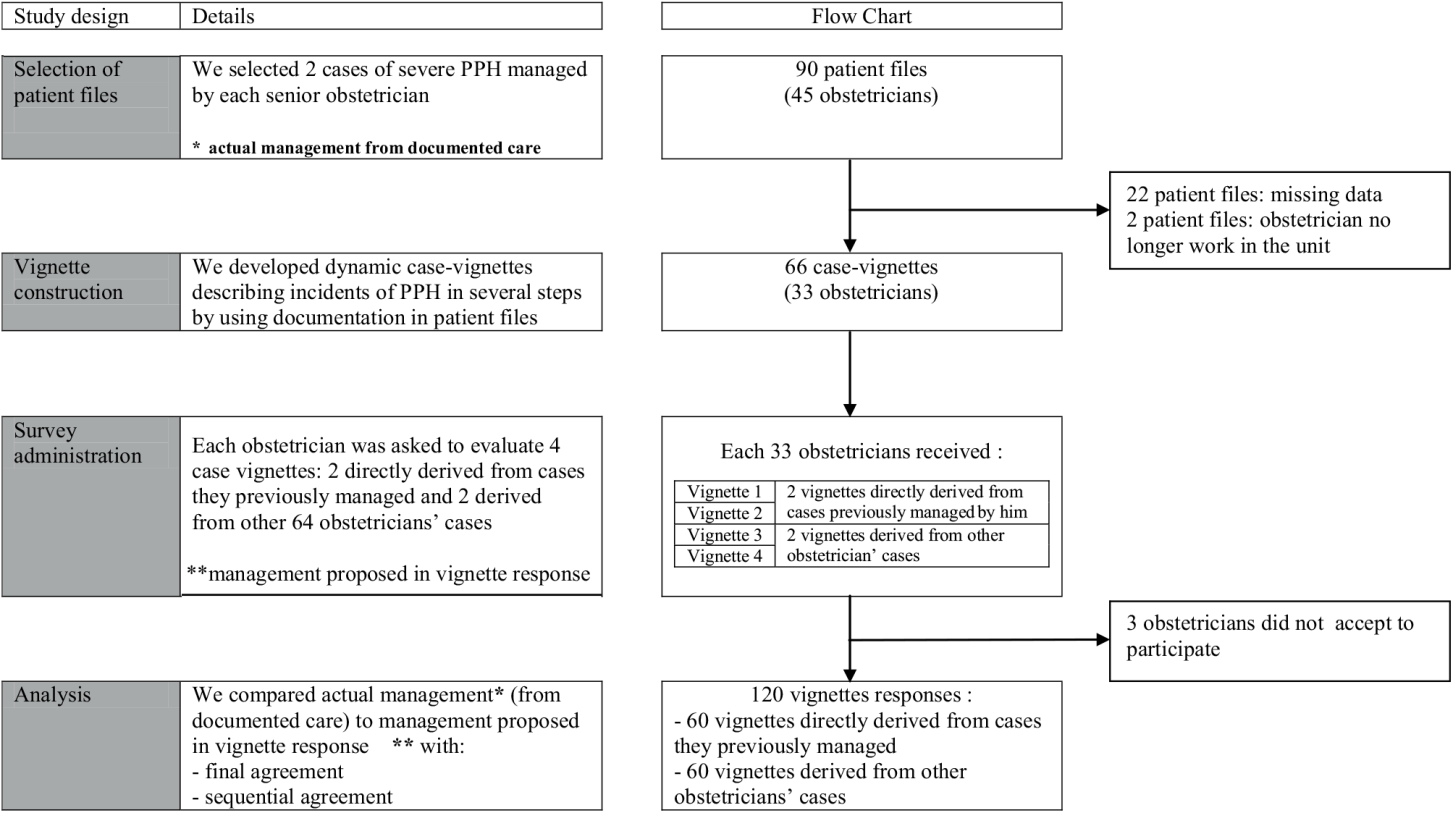
Study design. This figure describes study design and flow chart of the study.

Of the 33 obstetricians we contacted, 30 participated. Each evaluated 4 vignettes, for a total of 120. Most participants worked in university hospitals (n = 27). (Fig 5)

### Main Outcomes: Agreement between Vignette Responses and Documented Care

#### Final agreement

In cases each obstetrician had previously managed, agreement between final treatment decisions in vignette responses and documented care was substantial, with a Kappa coefficient of 0.75 (95% CI [0.62–0.88]). Overall agreement was 49/60 (82%). (Table 1)

**Table 1:** Final agreement in cases previously managed by the obstetrician.

	Final management proposed in vignette response				
Final actual management	Sulprostone	Tamponade	Embolization	Surgery	Hysterectomy
Sulprostone	1	0	0	0	0
Tamponade	0	14	1	0	0
Embolization	1	2	17	2	0
Surgery	0	0	1	7	2
Hysterectomy	0	0	0	2	10

On the other hand, agreement between final treatment decisions in vignette responses and documented care for the cases the obstetricians had not previously managed themselves was only fair, with a Kappa coefficient of 0.30 (95% CI [0.12–0.48]). Overall agreement was 29/60 (48%). (Table 2)

**Table 2:** Final agreement in cases managed by other obstetrician.

	Final management proposed in vignette response				
Final actual management	Sulprostone	Tamponade	Embolization	Surgery	Hysterectomy
sulprostone	1	0	0	0	0
Tamponade	0	5	9	1	0
Embolization	0	7	10	4	1
Surgery	0	1	3	5	2
Hysterectomy	0	0	1	2	8

Final agreement between cases previously managed and cases managed by other obstetrician differed significantly. Overall agreement was significantly better for cases corresponding to situations the obstetrician had previously managed than situations managed by others (82% versus 48%, p<0.001).

#### Sequential agreement

In the group of cases previously managed by the obstetrician, sequential agreement between treatment decisions in vignette responses and documented care for the series of steps was high for 43/60 vignettes (72%). Among the 17 cases with low agreement, five involved hysterectomies performed immediately in actual practice but delayed in the vignette response. In five other cases, the vignette response chose intrauterine tamponade, which was not used in actual practice. Finally, in the last 7 cases, the proposed management in the vignette response was very dissimilar from the actual practice.

For cases the obstetricians had not previously managed themselves, sequential agreement between vignette responses and documented care was high for substantially fewer vignettes (25/60 vignettes, 42%). Thus the sequential agreement was significantly better for situations the obstetrician had managed before than for situations based on the cases of other obstetricians (72% versus 42%, p = 0.002).

Importantly, among the 120 situations analyzed by consensus, the inter-rater agreement between the 2 specialists was almost perfect, with a Kappa coefficient of 0.86 [0.77–0.95].

#### Responses to additional questions

To the question about whether they thought these vignettes were derived from real cases, obstetricians answered yes for 90/120 situations (75%), no in 8 (7%), and perhaps in 22 (18%).

Only one of the 30 obstetricians accurately selected his or her own two cases; 26/30 selected 1 of their previous cases, and 2/30 selected none of their own previous cases. Finally, one selected all 4 cases as his own.

The final question was whether these obstetricians thought they had changed their PPH management over the past 2 years. Twenty thought they had, and nine that they had not. One did not answer the question.

## Discussion

### Principal Findings of the Study

Our study shows that dynamic vignettes are a valid tool that can accurately reflect real practices in complex emergency situations such as severe PPH. Indeed when obstetricians had previously managed a case identical to that described in the vignette, final agreement between treatment decisions in vignette responses and the documented care was substantial, and sequential agreement was significant. Furthermore, most often, obstetricians did not reproduce the strategies used by other obstetricians. They reproduced what they actually do, and responses to dynamic vignettes reflect their actual practices in PPH management.

### Clinical Meaning of the Study

Postpartum hemorrhage represents a common emergency situation necessitating a complex strategy. Our dynamic vignettes took this complexity into account. Policies for management of PPH vary both between countries and between maternity units within countries [24–27]. Our study confirms the considerable variability in practices for PPH management, for we found only fair agreement between another obstetrician’s actual final management as documented in the source record and the respondent physician’s final management in vignette responses.

Peabody et al [7,8] compared scores for quality of practices measured by vignettes, chart abstraction, and standardized patients. They concluded that quality of health care can be measured in an outpatient setting by using clinical vignettes. Also Baldwin et al [5] used clinical vignettes to assess physicians’ adherence to guidelines for ovarian cancer screening. Before dynamic vignettes can be used to measure quality of practice in complex emergency management, it is necessary to validate whether they accurately reflect what physicians do in actual practice in this context. Our study, which has done so, was thus a necessary prerequisite to their use for describing and assessing management in complex emergency situations.

### Strengths and Limitations of the Study

The main strength of the study is the originality of the vignette design involving several steps; this made it possible to examine management in complex emergency situations.

However, even though dynamic vignettes included several steps to re-create management of emergencies, this approach has limitations. Clinical management is difficult to divide into several steps, and this segregation is partly artificial. The successive steps we defined could bias responses: a specific therapy might have been reported at the end of the first step or at the beginning of the second step. For instance in our study, some obstetricians used sulprostone and then intrauterine tamponade in actual clinical practice but proposed sulprostone and intrauterine tamponade simultaneously in the vignette. To limit this bias, we qualitatively tested sequential agreement by consensus.

Potential memory bias was reduced by leaving at least a one-year interval between the date of obstetrician handled the case (2010–2011) and the survey (2012) and by changing vignette data that might identify the specific situation. Unfortunately this delay also modified agreement because practices have changed in France in the interim. For example, of the 17 cases previously managed for which sequential agreement was low, five involved intrauterine tamponade, a practice that was introduced only recently and became widely accepted between the time period for which we selected files for vignettes (2010–2011) and the date of the survey (2012).[28,29] This recent change in practice probably resulted in an underestimation of the validity and replicability of vignettes.

The theoretical approach of the vignette is the main limitation of the case vignette method. The urgency and stress generated by PPH cannot be fully represented in the vignette. In our results, for example, hysterectomy was performed immediately in actual practice, but was delayed for trial of other management in 5 vignette responses.

Finally, another limitation was the likely social desirability bias. Participants might have proposed, for example, what they know the clinical practice guidelines recommend (e.g., hysterectomy is a last choice) to present themselves in the best possible light. This social desirability bias probably explains some differences between management proposed in case-vignettes and in documented care.

## Conclusion

In conclusion, dynamic vignettes with several steps are a reliable tool for assessing actual practices in complex emergency situation.

Therefore they may be used to assess the quality of management of complex emergency situations. They may be also used to understand the variability and variations in emergency practices and to identify factors associated with them.

## Supporting Information

S1 Table Database of vignette responses and documented care. (XLS)
